# Supplementary material for: The Ku subunit of telomerase binds Sir4 to recruit telomerase to lengthen telomeres in S. cerevisiae
Source: eLife. 2015 Jul 28;4:e07750. doi: 10.7554/eLife.07750 (PMC4547093; doi:10.7554/eLife.07750)
Supplement: Supplementary file 1. — (A) Yeast strains used in this study. (B) Plasmids used in this study. DOI: http://dx.doi.org/10.7554/eLife.07750.015 [file elife07750s001.docx]

**Supplementary File 1A: Yeast strains used in this study.**

| Strain Name | Description of genotype | Origin | Use in Figures |
| --- | --- | --- | --- |
| TCy43 | *MAT*a *ura3-53 lys2-801 ade2-101 trp1-1 his3-*∆*200 leu2-*∆*1 VR::ADE2-TEL adh4::URA3-TEL tlc1::LEU2 rad52::HIS3 pTLC1-LYS2-CEN* | Seto *et al*., 1999 | All conditions in Figure 3A. |
| TCy127 | *MAT*a*, ADE2, his3*∆*, leu2*∆*0, lys2*∆*0, trp1*∆*63, ura3*∆*0, RAD52, tlc1::Kan^R^, pTLC1-URA3* | Mozdy and Cech, 2006 | *SIR4* strain in Figures 2A, 3B, and Figure 3 – figure supplement 2. All conditions in Figure 3 – figure supplement 1. No tag control in Figure 5. No MS2CP tag strain in Figure 7. |
| yDZ460 | BY4741 *trp1::CgLEU2, tlc1::HisMX6, sir2::KanMX4, pTLC1-URA3-CEN* | This study | *sir2*∆ strain in Figure 3C. |
| yDZ462 | BY4741 *trp1::CgLEU2, tlc1::HisMX6, sir3::KanMX4, pTLC1-URA3-CEN* | This study | *sir3*∆ strain in Figure 3C. |
| yDZ464 | BY4741 *trp1::CgLEU2, tlc1::HisMX6, sir4::KanMX4, pTLC1-URA3-CEN* | This study | *sir4*∆ strain in Figure 3C. |
| yDZ466 | BY4741 *trp1::CgLEU2, tlc1::HisMX6, rif1::KanMX4, pTLC1-URA3-CEN* | This study | *rif1*∆ strain in Figure 3C. |
| yDZ468 | BY4741 *trp1::CgLEU2, tlc1::HisMX6, rif2::KanMX4, pTLC1-URA3-CEN* | This study | *rif2*∆ strain in Figure 3C. |
| yDZ470 | BY4741 *trp1::CgLEU2, tlc1::HisMX6, pTLC1-URA3-CEN* | This study | Wild-type strain in Figure 3C. |
| yDZ477 | TCy127 *with SIR3-gly8-(MS2CP)2::His3MX6* | This study | Sir3-(MS2CP)_2_ strain in Figure 7. |
| yDZ478 | TCy127 with *SIR4-gly8-(MS2CP)2::His3MX6* | This study | Sir4-(MS2CP)_2_ strain in Figure 7. |
| yDZ502 | TCy127 with *sir4::CgLEU2* | This study | *sir4*∆ strain in Figures 2A, 3B, and Figure 3 – figure supplement 2. |
| yDZ503 | TCy127 with *EST2-gly8-myc13::LYS2* | This study | *SIR4* strain in Figure 5. |
| yDZ516 | yDZ503 with *sir4::CgLEU2* | This study | *sir4*∆ strain in Figure 5. |
| yDZ536 | W303dip *sir4::CgLEU2/SIR4, yku80::His3MX6/YKU80* | This study | None |
| yDZ544 | W303-1a *SIR4 YKU80* spore derived from yDZ536 | This study | Wild-type strain in Figure 2B. |
| yDZ545 | W303-1b *sir4::CgLEU2 YKU80* spore derived from yDZ536 | This study | *sir4*∆ strain in Figure 2B. |
| yDZ546 | W303-1a *SIR4 yku80::His3MX6* spore derived from yDZ536 | This study | *yku80*∆ strain in Figure 2B. |
| yDZ547 | W303-1b *sir4::CgLEU2 yku80::*His3MX6 spore derived from yDZ536 | This study | *sir4*∆*yku80*∆ strain in Figure 2B. |
| yDZ559 | yDZ503 with *sir2::CgLEU2* | This study | *sir2*∆ strain in Figure 5. |
| yDZ560 | yDZ503 with *sir3::CgLEU2* | This study | *sir3*∆ strain in Figure 5. |
| yDZ563 | TCy127 with *SIR4-gly8-myc13::LYS2* | This study | Wild-type strain in Figure 6. |
| yDZ564 | yDZ563 with *sir2::CgLEU2* | This study | *sir2*∆ strain in Figure 6. |
| yDZ565 | yDZ563 with *sir3::CgLEU2* | This study | *sir3*∆ strain in Figure 6. |
| yDZ566 | yDZ563 with *rif1::CgLEU2* | This study | *rif1*∆ strain in Figure 6. |
| yDZ567 | yDZ563 with *rif2::CgLEU2* | This study | *rif2*∆ strain in Figure 6. |

**Supplementary File 1B: Plasmids used in this study.**

| Plasmid  Name | Description of genotype | Origin | Use in Figures |
| --- | --- | --- | --- |
| pSD107 | *TLC1* in a pRS414 plasmid backbone | Diede and Gottschling, 1999 | *TLC1* in Figures 2, 3, 5, and 7. |
| pRS414 | *pTRP1-CEN vector* | Sikorski and Hieter, 1989 | *tlc1*∆ in Figure 5. |
| pDZ154 | *tlc1*∆*48* in a pSD107 plasmid backbone | Zappulla *et al*., 2011 | *tlc1*∆48 in Figures 2, 3, 5, and 7. |
| pKG274 | *TLC1(Ku)_3_* in a pSD107 plasmid backbone | This study | *TLC1(Ku)_3_* in Figures 2, 3, 5, and 7. |
| pDZ641 | *TLC1(MS2)_10_* in a pSD107 plasmid backbone | Lebo *et al*., 2015 | *TLC1(MS2)_10_* in Figure 7. |
| pDZ666 | *tlc1*∆*48(MS2)_10_* in a pSD107 plasmid backbone | This study | *tlc1*∆*48(MS2)_10_* in Figure 7. |
| pDZ930 | *SIR4* ORF in a pET-24d backbone. An extra methionine and glycine were added onto the N-terminus of Sir4 for cloning purposes*.* | This study | Used to express Sir4 in vitro in Figure 4. |
